# Le Dr Jean Languillon (1912-2003), un léprologue majeur trop méconnu

**DOI:** 10.48327/mtsi.v3i2.2023.379

**Published:** 2023-06-19

**Authors:** Jean Paul LOUIS, Francis LOUIS

**Affiliations:** 1 Diplômé de médecine tropicale, titulaire du CES de santé publique et de la spécialité de recherches du Service de santé des armées en lutte contre les grandes endémies.; 2 Spécialiste des hôpitaux des armées en biologie et diplômé de santé publique. Président-fondateur de l'Association des anciens et amis du Pharo, « Ceux du Pharo ».

**Keywords:** Jean Languillon, Léprologie, Institut Marchoux, Ordre de Malte, Afrique subsaharienne, Jean Languillon, Leprology, Institut Marchoux, Order of Malta, Sub-Saharan Africa

## Abstract

Disposant de documents inédits, les auteurs dressent le portrait d'un acteur essentiel dans la lutte contre la lèpre et rappellent – outre son *Précis de léprologie* qui reste la référence pour l'Afrique noire – ses apports majeurs, que ce soit sur l'origine immunologique de la maladie, la mise au point de schémas thérapeutiques efficaces ou la conception de stratégies pertinentes et efficaces de prise en compte du patient dans les différents aspects et complications de la maladie.

L'apport du Dr Languillon dans la lutte contre la lèpre ne peut se réduire au *Précis de léprologie* qui reste la référence pour tout intervenant sur cette maladie en Afrique noire. Ce serait oublier ses travaux établissant l'origine immunologique de la lèpre et de ses complications cutanées, réactionnelles et neurologiques. Autre apport majeur, l'importance de sa contribution au traitement médical de la maladie, en particulier par la mise au point de la polychimiothérapie (PCT), et qui a fait de l'Institut Marchoux de Bamako l'un des 5 centres mondiaux collaborateurs de l'OMS en matière de recherches cliniques en léprologie. Jean Languillon s'est également inscrit dans une prise en charge holistique de la maladie en créant le premier service de chirurgie de la lèpre et en recourant à la physiothérapie, à la prise en charge orthopédique par un appareillage adapté, à la réhabilitation sociale… Sans oublier les aspects préventifs des complications par la nécessaire dispensation régulière des traitements et le contrôle des malades dispersés sur de vastes territoires en créant un corps d'infirmiers contrôleurs lèpre et spécialistes de la maladie qui a procuré un appui essentiel au médecin chargé le plus souvent d'un immense secteur où le besoin d’être secondé était une évidence. Il terminera sa carrière africaine en mettant en place l'ILAD, Institut de léprologie appliquée de Dakar, qui va offrir la gamme complète de prise en charge telle qu'il l'a toujours préconisée. Au terme de ce long parcours professionnel, Jean Languillon ne pourra résister à l'appel de l'Ordre de Malte qui lui proposera de faire bénéficier de son expertise les différents pays dans lesquels l'Ordre est impliqué.

## Abstract (en)

Languillon's contribution to the control of leprosy cannot be reduced to the manual of leprology which remains the reference for anyone working on this disease in sub-Saharan Africa. This would mean forgetting his works that established the immunological origin of leprosy and its cutaneous, adverse and neurological complications.

Another major aspect, the importance of his contribution to the treatment of the disease, in particular through the development of polychimiotherapy (PCT), which has made Institut Marchoux in Bamako one of the five WHO's collaborating centers in the field of clinical research in leprosy.

Languillon was also involved in a holistic approach of the disease by creating the first leprosy surgery unit and implementing physiotherapy, orthopedic care with appropriate equipment, and social rehabilitation… without forgetting preventive aspects of complications through the necessary regular administration of treatments, and control of patients spread over wide territories, by creating a corps of leprosy nurse monitors and leprosy specialists. These will provide essential support to the doctor most often in charge of a huge sector where the need of assistance was obvious.

Languillon ended his African career by creating the ILAD, Institute of Applied Leprology in Dakar, which offers the full range of care, as he always advocated.

Finally, he did not resist the call of Order of Malta which offered him to share his expertise in the different countries where the Order was involved.

Il y a 20 ans, le Dr Languillon nous quittait après une carrière entièrement consacrée au service des lépreux. Ses travaux ont fait l'objet de nombreuses publications scientifiques mais, en dépit de leur importance, leur auteur reste peu ou mal connu. L'opportunité qui nous a été donnée de disposer d'une biographie exhaustive non publiée à destination uniquement familiale, permet, avec l'accord de ses ayants droit, de lui rendre l'hommage mérité en mettant en avant son apport majeur à la fois, en termes de connaissance de la maladie de Hansen mais aussi sur le plan de la lutte et de la prise en charge au quotidien dans ses différents aspects.

Jean Languillon (Fig. 1) est né le 6 mars 1912 à Amiens dans une famille d'industriels du textile où il était clair qu'il serait ingénieur et prendrait la succession paternelle. Mais le jeune homme est davantage attiré par la chose militaire, l'histoire naturelle, la médecine, le goût des voyages et de la découverte, sans toutefois voir clairement quel pourrait être son avenir professionnel. D'une discussion fortuite avec un de ses amis qui lui fait part de sa vie future de médecin des troupes coloniales, lui apparaît un métier qui correspond exactement à ses « vocations cachées ». Au grand dam de sa famille, après deux années de préparation de médecine à Rochefort, il le concrétise en 1934 en intégrant l’école de santé militaire de Lyon qui possède une section « coloniale » [6].

**Figure 1:**
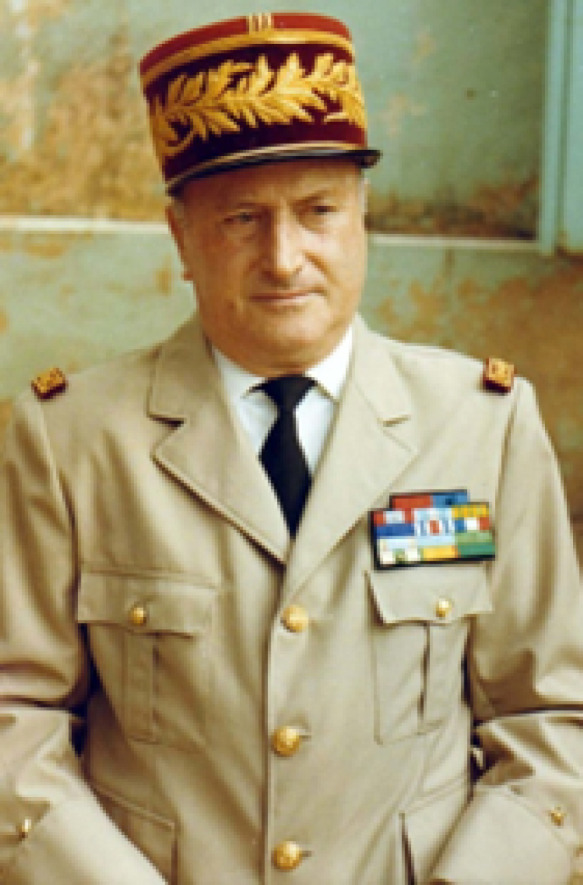
Jean Languillon (crédit photo : collection privée de l'auteur) Jean Languillon (photo credit: private collection of the author)

Reçu docteur en médecine en juin 1938, il effectue un bref séjour dans un régiment métropolitain avant d'intégrer l’École d'application des troupes coloniales du Pharo à Marseille. Mais, déclaration de guerre oblige, il est rapidement envoyé sur le front nord avec le 52^e^ régiment d'infanterie coloniale. Il y reste peu de temps, avant d’être affecté comme Médecin et Administrateur des îles du Centre des Nouvelles-Hébrides (19401943). C'est le début de sa véritable carrière de médecin colonial qu'il va poursuivre en occupant successivement les postes de Médecin et Résident de France aux îles Loyauté de Nouvelle-Calédonie (1944), où il commence à s'intéresser aux lépreux et aux recherches sur la sérologie de la lèpre, avant d'occuper le poste de Directeur de l'Institut Gaston Bourret à Nouméa (1945-1947) avec une implication forte dans la léproserie de Ducos qui centralise l'ensemble des lépreux dépistés sur l’île. Il est ensuite admis à l'Institut Pasteur de Paris pour y suivre le « grand cours » et, après une spécialisation en biologie, il est nommé Directeur de l'Institut Pasteur de Pointe-à-Pitre (1949-1951), affectation où il expérimente la Disulone, un nouvel antilépreux qui vient de sortir. Il est ensuite Médecin chef du centre de transfusion des Forces françaises d'Extrême-Orient à Saïgon (1952-1954) et enfin Chef du Service antipaludique et Directeur de l'Institut de recherches médicales du Cameroun qu'il a créé en attendant sa transformation envisagée en Institut Pasteur de Yaoundé (1955-1957), affectations à l'issue desquelles débute réellement la carrière de médecin léprologue qui fera sa renommée.

À peine rentré en France, il est appelé, sur demande du Médecin général Richet, Directeur des grandes endémies en Afrique francophone, à prendre le poste de Directeur de l'Institut Marchoux à Bamako. C'est là, dit Languillon, qu'après la traditionnelle visite à Dakar à Richet, son supérieur hiérarchique, que, de retour à Bamako, impressionné par le charisme et l'appui obtenu de ce grand tropicaliste, « je décidai de devenir un lépro-logue de renommée internationale » (Fig. 2). Quoique pasteurien, biologiste, paludologue, entomologiste, hématologue, il dit lui-même : « Je ne savais pratiquement rien sur la lèpre, je n’étais ni léprologue ni dermatologue. » Cependant, il comble rapidement ces lacunes au prix d'un travail acharné.

**Figure 2:**
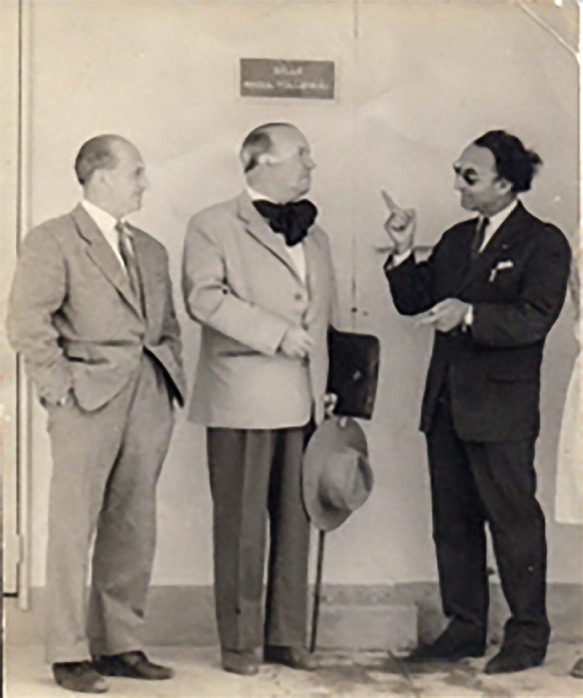
De gauche à droite : Jean Languillon, Raoul Follereau et Pierre Richet à l'institut Marchoux (crédit photo : collection privée de l'auteur) From left to right: Jean Languillon, Raoul Follereau and Pierre Richet at the Marchoux Institute (photo credit: private collection of the author)

Pour mémoire, en 1930, la « Société spéciale de la lèpre » de la Société des Nations, réunie à Bangkok, avait retenu l'internationalisation de la lutte contre la lèpre en adoptant des règles communes d'action et en standardisant les conduites thérapeutiques. Dans la foulée de ces orientations, Émile Marchoux, pionnier de l'approche moderne du traitement de la lèpre, crée en 1931, à Bamako, alors capitale du Soudan français, le Service de prophylaxie de la lèpre, lequel deviendra en 1935 l'Institut central de la lèpre avant de prendre le nom d'Institut Marchoux en 1945, avec pour vocation d’être à la fois centre de lutte contre la lèpre, centre de recherche clinique et thérapeutique et centre de formation [2]. Languillon, dont le séjour devait durer 2 ans, y restera en fait de 1957 à 1972.

Il y développe son action et ses travaux dans plusieurs directions en établissant en particulier l'origine immunologique de la lèpre et de ses complications cutanées, réactionnelles et neurologiques.

Aidé par un jeune chirurgien, le Dr Bourrel, il crée le premier service de chirurgie de la lèpre où son confrère développe son expertise qui le rendra célèbre dans la chirurgie de la main lépreuse (Fig. 3). Autre chirurgien de renommée internationale, André Carayon y prend également sa part, notamment pour ce qui est de la thérapeutique chirurgicale des troubles trophiques de la maladie de Hansen. Tous ces travaux feront l'objet d'un ouvrage de référence consacré à la chirurgie de la lèpre [1]. Outre la chirurgie réparatrice, il convient de mentionner la mise en place de la physiothérapie, la prise en charge orthopédique par un appareillage adapté fabriqué sur place dans un atelier *ad hoc* et la réhabilitation sociale par le biais d'une école dirigée par un instituteur lui-même lépreux, toutes approches novatrices dans la prise en charge de la maladie.

**Figure 3:**
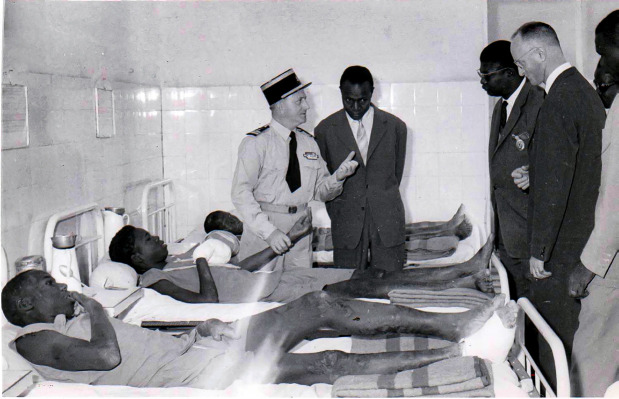
Languillon au chevet des maladesà Marchoux, présentant la maladie à ses visiteurs (crédit photo : collection privée de l'auteur) Languillon at the beside of the sicks in Marchoux, presenting the diseaseto visitors (photo credit: private collection of the author)

Autre apport majeur, l'importance de la contribution apportée par Languillon au traitement médical de cette maladie : chaque nouveau produit anti-hansénien prôné de par le monde est testé à l'Institut Marchoux qui devient l'un des 5 centres mondiaux collaborateurs de l'OMS en matière de recherches cliniques en léprologie. Plus de 20 médicaments, dont plusieurs envoyés par l'OMS, notamment ceux utilisables *per os* hebdomadairement et donc utilisables en campagnes de masse, vont y être testés avec toute la rigueur méthodologique qui s'impose. Ces recherches sont facilitées par la présence en périphérie de Bamako du village de postcure de Djikoroni fondé par Languillon – sur financement en particulier de l'Association Raoul Follereau – centre qui permettait de traiter des malades venus de tout l'Ouest africain et d'en suivre le résultat sur le long terme. Sans oublier les essais en vraie grandeur comme cela a été le cas par exemple dès 1965, avec la mise en place d'un circuit à la Kelfizine dans le Cercle de Diré au Mali.

On peut retenir en particulier ses travaux sur un sulfamide, le Sultirène, sur les sulfamides retard, Fanasil et Kelfizine, sur la clofazimine (Lamprène) active sur les formes résistantes, sur l'association sulfone + clofazimine ainsi que sur un antibiotique, la Rifampicine en montrant son efficacité avec une dose mensuelle administrée pendant 6 mois. Il en découlera la PCT associant ces trois médicaments qui constitue toujours le traitement de référence de l'OMS (traitement de première ligne ou en première intention) par administration mensuelle avec supervision médicalisée. Plus tard, alors que Languillon a quitté le Mali, l'apparition des fluoroquinolones lui donnera l'occasion d’étudier l'Ofloxacine et la Minocycline et de préconiser l'association R+O+M (Rifampicine + Ofloxacine + Minocycline) en dose unique chez les lépreux tuberculoïdes et en prise mensuelle chez les patients interpolaires et les lépromateux.

Autre apport majeur de Languillon, l'enseignement qui a occupé une part importante de son activité et où, de l'avis unanime, il s'est montré un excellent pédagogue. Il crée en particulier, en 1960, le corps des infirmiers contrôleurs lèpre. Pour bien saisir l'importance de cette initiative, un petit rappel historique s'impose.

Dès sa création, le service de santé aux colonies avait recommandé les tournées médicales comme l'une des activités essentielles du médecin en poste outre-mer. Cette médecine mobile a été mise en œuvre dès 1917 par Eugène Jamot dans le cadre de la lutte contre la maladie du sommeil. En 1944, devant les bons résultats obtenus par cette méthode, le médecin général Marcel Vaucel a proposé de l’étendre à d'autres maladies endémiques comme la lèpre et a mis en place le Service général d'hygiène mobile et de prophylaxie (SGHMP) d'abord en Afrique-Occidentale française puis en Afrique-Équatoriale française.

En 1955 à Bobo Dioulasso, en Haute-Volta, le médecin colonel Richet développe le concept et bâtit le Centre de référence Muraz, institution névralgique dotée de laboratoires bien équipés où travaillent médecins et chercheurs appuyés par un centre de documentation adapté. Est simultanément créée l’École Jamot, qui forme tous les ans plus de 100 infirmiers destinés aux différents secteurs du SGHMP [5].

Avec l'aide du jeune médecin René Labusquière, Pierre Richet met en place une méthode originale de distribution de la Disulone aux lépreux, les circuits « marguerite », tournées cyclistes quotidiennes au cours desquelles un aide-infirmier parcourt un circuit en « pétale de fleur » qui le ramène chaque soir à son point de départ et repasse deux fois par mois dans chaque village. Cette stratégie a perduré avec le soutien de l'UNICEF et de Raoul Follereau qui fournissent médicaments, bicyclettes et véhicules si nécessaire.

C'est dans cette continuité que, sur la base d'un stage trimestriel, Languillon forme annuellement 25 infirmiers du Service des grandes endémies pour en faire des « contrôleurs lèpre » chargés du dépistage et du contrôle de traitement des malades dans le cadre des circuits en « marguerite », en complément de la dispensation assurée par les aide-infirmiers distributeurs de médicaments. Dès 1965 y est ajouté le corps des infirmiers spécialistes de la lèpre au cours d'un stage étalé sur un an. Ces infirmiers contrôleurs lèpre et spécialistes de la lèpre vont apporter un appui essentiel au médecin chargé le plus souvent d'un immense secteur où le besoin d’être secondé est une évidence. Des « Comptes rendus trimestriels de l'Institut Marchoux » seront diffusés de 1960 à 1972 à l'ensemble des médecins de secteurs et des infirmiers impliqués dans la lutte contre la lèpre.

Ces travaux donneront lieu à 175 publications et surtout, en 1969, au *Précis de léprologie* [4], ouvrage de référence pour tous les acteurs de la lutte contre la maladie hansénienne ou qui voudraient s'y intéresser et auquel Richet rend ainsi hommage : « La lecture de cet ouvrage m'a captivé, passionné. J'ai eu l'impression de “possession” du sujet pour tous les chapitres, c'est bien le meilleur test de la valeur didactique de l'ouvrage tout entier. »

En 1972, au terme de son séjour au Mali, Languillon est approché par l'Ordre de Malte avec la proposition de créer à Dakar un Institut de léprologie subventionné par cet Ordre avec l'approbation du Président Senghor, proposition assortie de la direction d'une chaire de léprologie, nouvelle étape majeure dans son cursus scientifique et fin de sa carrière militaire marquée par l'accession au grade de médecin général.

Suivant les plans de Languillon, l'ILAD, Institut de léprologie appliquée de Dakar, offre une gamme complète de prise en charge avec un pavillon d'hospitalisation, un pavillon chirurgical, un pavillon de physio-ergothé-rapie et de prothèse, un laboratoire, un pavillon pour les consultations, la bibliothèque et l'enseignement, ainsi que divers locaux de pharmacie, réfectoire, lingerie… (Fig. 4).

**Figure 4:**
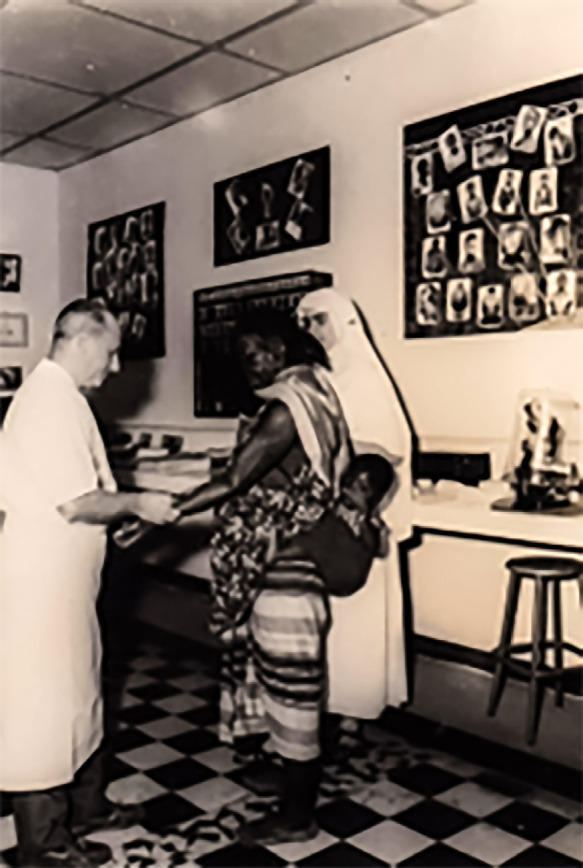
Jean Languillon en consultation à l'ILAD (crédit photo : collection privée de l'auteur) Jean Languillon in consultation at the ILAD (photo credit: private collection of the author)

Quatre orientations principales vont y être développées:

1. Traitements médicaux, chirurgicaux et de réhabilitation, facilités par l'architecture adaptée de l'Institut;
2. Recherches d'ordre thérapeutique selon les formes cliniques : Clofazimine, sulfone retard (DADDS), Rifampicine utilisée seule en traitement mensuel . qui vont faire l'objet de plus de 40 communications;
3. Santé publique en tant que Conseiller de la lutte contre la lèpre auprès du directeur du Service des grandes endémies avec en particulier la création d'une zone pilote dans une région d'hyper-endémie retenue comme terrain de stage et de formation pour le personnel médical et paramédical (Fig. 5);
4. Enseignement dans différentes structures au titre de Professeur associé de léprologie de la faculté de médecine de Dakar : enseignement universitaire et post-universitaire médical et paramédical, Institut de médecine tropicale de Dakar, Institut de médecine tropicale de Paris, organisation d'un cours OMS de léprologie en langue française dispensé à l’étranger, entre autres à Addis-Abeba et à Papeete, poursuite de la formation des infirmiers « lèpre » à l'instar de ce qui se passe au Mali.

**Figure 5:**
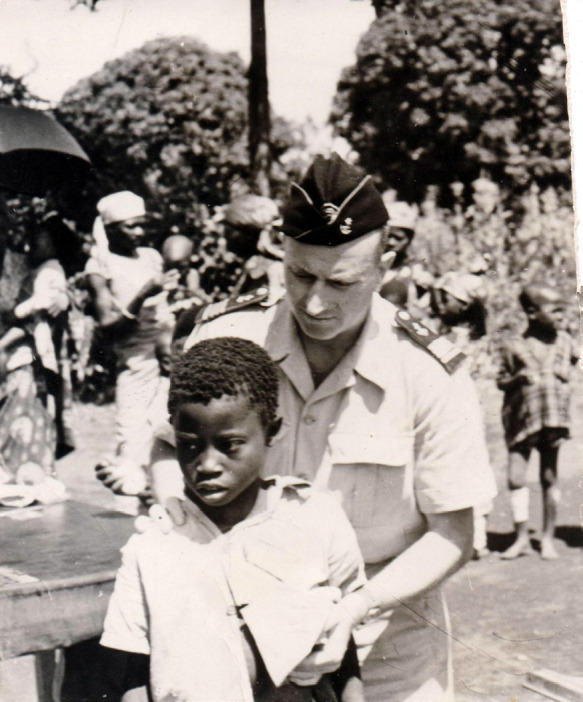
Dépistage de la lèpre par Languillon dans un village sénégalais (crédit photo : collection privée de l'auteur) Screening of leprosy in a Senegalese village (photo credit: private collection of the author)

Nommé Vice-président de l'Association internationale de léprologie puis Président de l'Association des léprologues de langue française, Languillon met un terme à son engagement au Sénégal en 1977 et prend ce qu'il pensait être sa retraite.

Il compte alors revenir à certaines de ses « vocations cachées » d'antan – la géologie, la paléontologie, la vulcanologie – et envisage même de « faire une maîtrise et pourquoi pas un doctorat ».

Mais ses consultations et ses cours lui manquent et il ne va pas tarder à reprendre ses activités par le biais de l'Ordre de Malte qui lui propose de faire bénéficier de son expertise les différents pays dans lesquels l'Ordre est impliqué. C'est ainsi que, de 1979 à 1995, il interviendra en tant que Conseiller technique en Somalie, au Brésil (plus de 20 missions), au Paraguay, en Uruguay (4 missions), en Tunisie (10 missions) où, indépendamment de son appui dans l'organisation de la lutte, il assure à maintes reprises des cours de léprologie.

En 1995, bardé de titres universitaires et de multiples décorations, membre d'une dizaine de sociétés savantes françaises et étrangères, il se retire définitivement dans sa maison de Corse où il publie en 1999 une édition superbement illustrée et actualisée de son *Précis de léprologie* prenant en particulier en compte l’évolution des connaissances sur l'immunologie de la maladie. Il élabore cet ouvrage en collaboration avec des spécialistes tout aussi prestigieux, comme lui anciens médecins « coloniaux » ayant eu l'occasion de s'intéresser à la lèpre au cours de leurs séjours outre-mer : P. Bourrel pour la chirurgie de la lèpre, G. Discamps pour l'anatomopathologie, P. Saint-André pour le diagnostic différentiel des lésions cutanées hanséniennes et G. Baquillon pour les questions liées à l'identification de *Mycobacterium leprae* [3].

Il décède paisiblement auprès des siens en 2003 après une vie bien remplie, entièrement consacrée au service des lépreux.

## Liens D'intérêts

Les auteurs ne déclarent aucun lien d'intérêt.
